# Investigating Web-Based Nutrition Education Interventions for Promoting Sustainable and Healthy Diets in Young Adults: A Systematic Literature Review

**DOI:** 10.3390/ijerph19031691

**Published:** 2022-02-01

**Authors:** Nadine Ghammachi, Putu Novi Arfirsta Dharmayani, Seema Mihrshahi, Rimante Ronto

**Affiliations:** Department of Health Sciences, Faculty of Medicine, Health and Human Sciences, Macquarie University, Sydney, NSW 2109, Australia; putu-novi-arfirsta.dharmaya@hdr.mq.edu.au (P.N.A.D.); seema.mihrshahi@mq.edu.au (S.M.); rimante.ronto@mq.edu.au (R.R.)

**Keywords:** young adults, web-based interventions, sustainable and healthy diet

## Abstract

Background: Our current rapidly growing food systems are imposing a heavy burden on both environmental sustainability and human health. Sustainable and healthy diets aim to promote optimal health and have a minimal environmental impact. This study aimed to critically review and synthesise the evidence on the effectiveness of web-based nutrition education interventions aiming to promote sustainable and healthy diets among young adults. Methods: A systematic search of four databases (Medline, PsycINFO, Scopus, and Embase) was conducted in March 2021. Studies were included if they used an online platform to deliver the intervention to young adults and measured at least one aspect of sustainable and healthy diets, such as plant-based food intake, food waste, and local and seasonal produce. Of the 2991 studies, a total of 221 full-text articles were assessed for eligibility of which 22 were included in the final review. Results: A majority of the studies (82%) targeted fruit and vegetable consumption, and close to a quarter of studies (23%) targeted other aspects of a sustainable and healthy diet, such as red meat intake. Only one study included multiple aspects of a sustainable and healthy diet. Program delivery outcomes reported overall positive feedback and engagement. Conclusion: This review suggests that web-based interventions may be effective in promoting some sustainable diet-related outcomes in young adults. However, there is a need for developing and evaluating future programs to promote sustainable diets more comprehensively in order to help young adults make healthy and sustainable food choices.

## 1. Introduction

The rapidly growing food systems are currently imposing a heavy burden on both environmental sustainability and human health [1,2]. Evidence shows that unsustainable food systems at different stages of production, storage, transportation, and consumption, have a significant effect on greenhouse gas emissions (GHGE). Agricultural GHGE are one of the largest drivers for climate change and biodiversity degradation [1]. More specifically, food systems, agriculture, and our dietary behaviours contribute to 30% of GHGE and occupies around 40% of land and almost 70% of freshwater use [3]. A few years ago, the EAT-Lancet Commission on Healthy Diets from Sustainable Food Systems stated that there is a need to alter our current unhealthy and unsustainable diets to improve health and minimize the environmental impact [1]. To promote sustainable and healthy diets, it has been recommended to shift to plant-based food sources, local and seasonal produce, and minimally processed foods.

The World Health Organization (WHO) described sustainable and healthy diets as “healthy dietary patterns that aim to promote optimal health and wellbeing and have minimal environmental pressure and impact. Sustainable healthy diets are equitable, affordable, accessible and culturally acceptable” [4]. Uncertainty remains around the measurable components that constitute a sustainable and healthy diet. However, some measurable components of a sustainable diet have been identified in the literature: (1) reduction in overconsumption; (2) higher intake of plant-based foods (fruits, vegetables, grains, and legumes); (3) reduction in animal-derived and processed foods; (4) focus on local and seasonal produce; and (5) reduction in food wastage [1,5]. Existing literature shows that foods which have a minimal environmental impact, such as fruits, vegetables, and grains are also linked to better health outcomes than foods with larger environmental impacts, such as animal-derived foods and processed foods [3,6,7]. However, national nutrition data in Western countries indicates that most young adults consume less than the recommended servings of fruits, vegetables, and whole grains and overconsume processed and discretionary (energy-dense, nutrient-poor) foods [8,9,10,11]. It is important to promote healthy dietary behaviours among young adults, as often, poor dietary behaviours established early in life persist into adulthood and may increase the risk of noncommunicable diseases, such as diabetes, cardiovascular diseases, and certain cancers [9,10,12,13].

To address unhealthy dietary behaviours, there has been an increase in public health nutrition interventions aiming to promote healthy dietary behaviours among young adults [10]. A few systematic literature reviews synthesised scientific evidence on the effectiveness of interventions aiming to promote healthy dietary behaviours among young adults have shown some positive outcomes, such as changes in overall diet quality and increases in fruit and vegetable intake [9,10]. Despite positive changes in fruit and vegetable consumption, evidence was less significant for other dietary outcomes, such as energy-dense, nutrient-poor (EDNP) food intake [10]. In addition, there has been growing evidence regarding the use of web-based/online nutrition interventions to tackle unhealthy dietary behaviours, especially among young adults [14,15]. A recent systematic literature review suggested the use of web-based/online platforms as an effective delivery mode, considering the high engagement of young adults with these platforms [16]. However, there is limited evidence on the effectiveness of web-based interventions in promoting sustainable and healthy diets among young adults. Therefore, this study aimed to critically review and synthesise the literature on web-based interventions aiming to promote sustainable dietary behaviours among young adults.

## 2. Materials and Methods

This systematic literature review was guided by the Preferred Reporting Items for Systematic Reviews and Meta-Analyses reporting (PRISMA) statement [17], based on a predefined protocol developed by the research team and registered on PROSPERO (CRD42021239377). No changes have been made from the original protocol submission. A scientific database search was conducted searching the four following databases on the third of March 2021: Medline, Scopus, Embase, and PsycINFO via Ovid. These databases afford broad coverage of public health, health promotion, and nutrition literature. Then search terms were applied based on four main concepts: *sustainable diet, young adults, web-based, and intervention.* Appendix A contains a full list of search terms and proposed search strings that were used.

### 2.1. Eligibility Criteria

The target population was young healthy adults aged 18 to 25 years old. Studies that recruited young adults presenting with any noncommunicable disease or any other medical condition(s) that requires a specific diet were excluded. This review included randomised controlled trials, nonrandomised controlled trials, and studies with pre- and post-study designs. Studies were included in the review if the interventions were delivered via any web-based online platform (e.g., website, social media platforms, and mobile application). Outcomes of interest included at least one of the aspects of sustainable and healthy diets: (1) increase in plant-based food intake (e.g., fruit and vegetables, grains, legumes), (2) reduction in food waste in terms of over-consumption, (3) reduction in discretionary and ultraprocessed food intake (e.g., energy-dense nutrient poor (EDNP)), and/or 4) reduction in animal-derived food intake (e.g., red and processed meat intake). Interventions targeting weight loss or measuring overall diet score as a primary outcome were excluded from this review. Table 1 represents in detail the inclusion and exclusion criteria.

### 2.2. Selection Process

**Figure 1:**
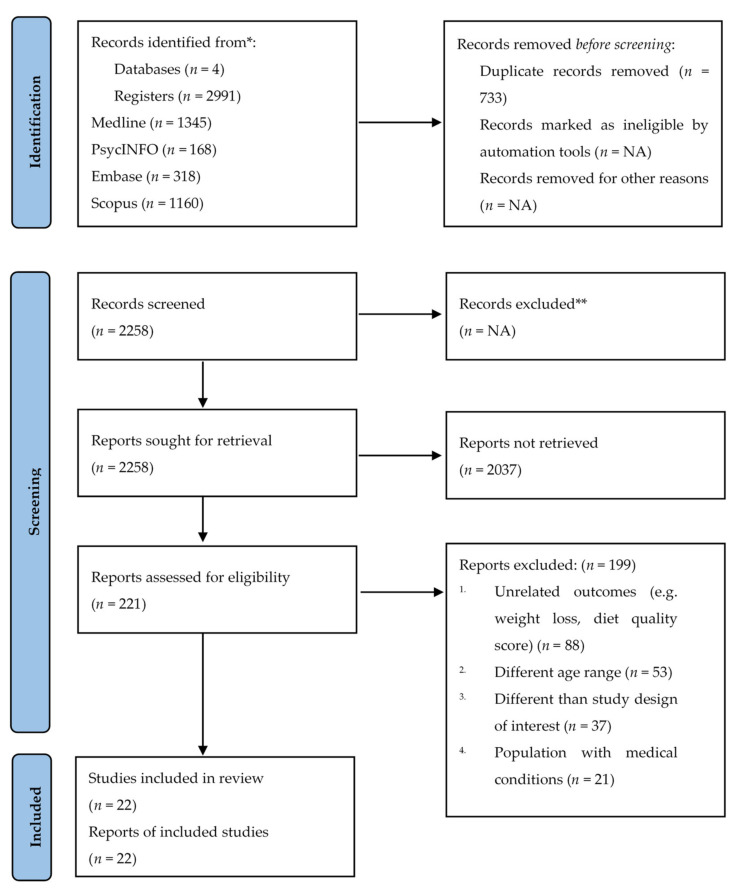
The PRISMA flow diagram showing the selection process of the included studies. * Consider, if feasible to do so, reporting the number of records identified from each database or register searched (rather than the total number across all databases/registers). ** If automation tools were used, indicate how many records were excluded by a human and how many were excluded by automation tools. NA: not applicable.

Figure 1 shows a PRISMA flow diagram of the article screening and selection process. The initial search was performed by one researcher (NG) through all four databases. All studies identified through the database searches were extracted and stored in Endnote v8 (Thomson Reuters 2017), then the duplicates were removed. Studies were then screened for suitability of inclusion in the review based on title, abstract, and keywords by one researcher (NG). The full-text screening was performed by two researchers (NG and PNAD) independently. All disagreements were resolved by a consultation with a third researcher (RR). The reference lists of all included studies were also hand searched for relevant studies not identified in the first search strategy.

### 2.3. Data Extraction and Analysis

The data extraction phase of the 22 articles was performed by two researchers (NG and PNAD) independently based on the template created by the research team, which included the following: author, date published, country of study, study design, participant characteristics (age, gender, educational level, etc.), inclusion and exclusion criteria, description of intervention, control conditions, outcomes measured, duration of intervention, and follow-up. All results were discussed in narrative form due to the heterogeneity in dietary outcomes and measurement tools.

### 2.4. Quality Assessment

The quality of the selected studies was appraised using the Effective Public Health Practice Project (EPHPP) quality assessment tool for quantitative studies [18]. This quality assessment tool provides a score ranging from “strong”, “moderate”, and “weak” based on eight different categories: selection bias, study design, confounders, blinding, data collection method, withdrawals and dropouts, intervention integrity, and analyses. Two researchers (NG and PNAD) assessed risk of bias of each included study independently. Any disagreements were resolved by consensus and by consultation with a third researcher (RR) (Appendix B).

## 3. Results

### 3.1. Search Results and Characteristics of Included Studies

A total of 2991 records were identified through the initial search of the four databases. After removing all duplicates, 2258 articles remained. Full-text screening included 221 articles of which 22 articles met the eligibility criteria and were included in the final review (Figure 1). The most common reason for exclusion of articles from our final review was because the outcome was related to/focused on weight loss.

Of the total 22 included studies, 17 were randomized control trials (RCT) [11,13,19,20,21,22,23,24,25,26,27,28,29,30,31,32,33] and five were pre- and post- design studies [12,34,35,36,37]. The study characteristics are represented in Supplementary Table S1, and the summary of study results is provided in Table S2. In total, there were 13,463 participants, and the majority of participants were females. The interventions were conducted in six different countries: thirteen studies were conducted in the United States of America [12,21,23,24,25,28,29,30,31,32,34,36,37], three in the United Kingdom [11,13,35], two in Australia [26,33], two in Italy [19,20], and one each in New Zealand [27] and China [22]. Of the participants, 83% (91% of studies) were university and/or college students recruited through their educational institutions. Studies were published between 2005 and 2019; 10 have been conducted in the last five years. A majority of the interventions (*n*= 13) have been delivered via websites [11,13,22,23,24,27,28,29,32,33,34,35,36], with the remainder delivered via text-messaging and/or emails (*n*= 6) [19,20,25,30,31,37], mobile applications (*n* = 2) [12,26], and one as a TV cooking show (*n* = 1) [21]. The length of interventions varied from 20–30 min to 6 months.

A majority of the dietary outcomes were measured using food frequency questionnaires (FFQ) (*n* = 9) [12,21,25,28,29,30,33,34,37]. Other measures were also used to assess dietary outcomes, such as specific nutrient/food item/dietary behaviour questionnaires (*n* = 5) [11,13,22,27,32], dietary recall, and food diary or record (*n* = 5) [19,20,26,31,35]. Two studies used multiple methods for dietary assessment [23,24].

### 3.2. Study Quality Assessment

Overall, the methodological quality of included studies was ‘strong’ for six studies [23,24,30,31,32,33], ‘moderate’ for nine studies [12,13,19,20,26,35,36,37], and seven studies were rated as ‘weak’ [11,21,22,25,27,28,29,34]. The most commonly observed quality-related issue was regarding the data collection tools validity and reliability.

### 3.3. Use of Behaviour Change Theory

A total of 17 out of 22 (77 %) interventions were designed based on one or more behaviour change theories [11,13,19,20,21,22,23,24,25,26,30,31,32,33,34,35,36,37]. The Transtheoretical Model (TTM) (*n* = 8) [23,24,25,30,32,34,36,37] was the most commonly applied behaviour change theory in addition to other theories such as Theory of Planned Behaviour (TPB) (*n* = 5) [11,13,19,20,33], the Social Cognitive Theory (SCT) (*n*= 3) [21,24,33], and the Self-Affirmation Theory [11,13,35]. A majority (83%) of the studies which employed a behaviour change theory in intervention development reported significant changes in at least one of the primary outcomes [13,19,20,22,23,24,25,26,30,31,32,33,35,36,37].

### 3.4. Sustainable and Healthy Diet-Related Outcomes

#### 3.4.1. Knowledge Regarding Sustainable Diets and Food Preparation Skills

Only two out of twenty-two studies measured knowledge and food preparation skills regarding sustainable and healthy diets [21,36]. The *“Good Grubbin”* study consisted of a series of four 15-min episodes of a cooking show that aimed to improve knowledge and attitudes towards cooking and consumption of fruits and vegetables [21]. The intervention resulted in significant positive effects on knowledge and cooking motivators in the intervention group compared to the control group. The second study, *“The Green Eating project”*, was a five-week intervention including four educational modules regarding sustainable dietary behaviours [36]. Knowledge about sustainable dietary behaviours was significantly improved from baseline to post-intervention in the intervention group [36].

#### 3.4.2. Sustainable Dietary Behaviours

Only one study, *“The Green Eating Project”,* targeted multiple aspects of sustainable diets, including eating local, minimizing waste, and increasing environmentally friendly protein consumption using a five-week intervention. The main aim of the study was to motivate young adults to adopt these sustainable and environmentally conscious eating behaviours, which were called *“Green Eating”* (GE) behaviours [36]. The intervention resulted in significant improvement in GE behaviours, such as choosing local produce more often and selecting “free range” labelled meats and poultry more frequently [36].

#### 3.4.3. Fruit and Vegetable Intake

A majority of the studies (*n* = 18, 82%) measured fruit and vegetable intake as a primary outcome. Fourteen studies (78%) showed a statistically significant fruit and/or vegetable intake improvement in the intervention group post-intervention [12,13,22,23,24,25,26,27,28,29,30,31,35,37]. Out of these fourteen studies, the majority of studies delivered interactive activities, learning modules, and health messages through tailored websites [13,22,23,24,27,28,29,35,37]. Two studies delivered a series of educational materials through e-mails [25,30], one study developed a mobile application [26], another used the combination of website and text messaging [31], and one study used phone texting [12].

Kerr et al. [26] promoted fruit and vegetable consumption through a mobile application “*mFR app”,* providing tailored dietary feedback and support messages to two experimental groups and one control. Interestingly, this study reported a significant increase in fruit intake in one intervention group and only vegetable intake in the other intervention group and control. O’Brien and Palfai [31] promoted attainment of the recommended fruit and vegetable servings through a website and added text messages, which also showed a positive increase in vegetable but not fruit intake.

Among the 14 studies that reported significant positive effects, fruit and vegetable intake was measured as servings per day in seven studies [12,13,23,26,30,35,37], as number of cups per day in two studies [24,25], and as servings based on national guidelines in meeting recommended intake in three studies [22,27,31]. The remaining two studies measured it as serving size and frequency of consumption over the past four weeks in one study [29] and as frequency of consumption in one study [28].

The studies that showed no significant improvement in fruit and vegetable intake (4 out of 18 studies) consisted of a TV cooking show [21], a series of online videos [34], a website [33], and a website with a mobile application [11]. Intervention lengths ranged between one to six months, and all interventions were based on one or more behaviour change theory.

#### 3.4.4. Self-Efficacy in Consuming Fruits and Vegetables

Self-efficacy in fruit and vegetable consumption was measured in half of the studies (*n* = 11) with a variety of tools/questionnaires developed by the researchers, and they showed positive effects (72.7%) [11,13,22,23,25,28,30,32,33,34,37]. However, there were variations in reporting self-efficacy. As self-efficacy was reported as a separate measured outcome (*n* = 7) from other social cognitive variables, four out of seven studies reported significant improvements towards increased self-efficacy in consuming fruits and vegetables among participants in the intervention group [23,28,34,37]. In the remaining studies (*n* = 4), self-efficacy was reported as a social cognitive variable with other indicators combined based on stage of change [22,25,30,32]. These studies consistently showed better progression to action or the maintenance stage in the intervention groups compared to the controls [22,25,30,32].

#### 3.4.5. Discretionary and Processed Food Intake

Only two studies measured energy-dense nutrient poor (EDNP) food intake, also defined as discretionary foods, and showed a statistically significant reduction of EDNP food intake in the intervention group [26,33]. The EATS (Eating Advice to Students) intervention was delivered via website, providing personalized feedback, tips, and strategies on dietary behaviours, and showed a significant decrease in EDNP food intake by 4.8% of total energy intake [33]. Whereas the study that used the mobile application included tailored dietary feedback in addition to text messaging support and resulted in a significant reduction by 0.8 ± 0.2 servings of EDNP foods per day in the intervention groups compared with the controls [26].

#### 3.4.6. Red Meat Intake

Two studies promoted reduction of red meat and processed meat consumption through text messaging [19,20]. There was a statistically significant difference between the intervention and control groups in red meat and processed meat consumption in both studies. In one study, the intervention group consumed significantly less portions of processed meat (mean= 1.74 portion/week) than the control group (mean = 3.29 portion/week) [19]. Similarly, in the second study, participants in the intervention group reported a lower consumption of red meat (mean= 1.62 portion/week) compared to the controls (mean= 3.03 portion/week) [20]. Both studies reported a statistically significant increase in positive intentions and attitudes towards reducing red meat and processed meat intake [19,20].

### 3.5. Program Delivery: Feedback and Participant Engagement

Less than half of the included studies (*n* = 8, 36%) reported participant engagement with the program and feedback or involved some form of process evaluation [11,12,13,21,25,32,33,36]. Feedback was provided by participants regarding overall enjoyment, satisfaction, usefulness, whether they recommend the program to others, applicability, and insights on task completion and engagement. A majority of the studies (*n* = 6) reported positive participant feedback about the interventions [12,21,25,32,33,36]. Two studies reported low engagement with the intervention [11,13].

## 4. Discussion

This systematic literature review is the first to explore sustainable dietary outcomes in web-based interventions among young adults. Of the 22 included studies, 20 (90.9%) showed encouraging results with respect to certain sustainable diet-related practices, such as increase of fruit and vegetable intake, reduced intake of processed foods and red meat, self-efficacy in improving sustainable dietary outcomes, and knowledge and attitudes towards sustainable dietary behaviours. This suggests that web-based interventions may be effective in promoting some sustainable diet-related outcomes in young adults, in particular, an increase in fruit and vegetable intake.

This review found only one study of moderate quality that targeted the majority of measurable outcomes of sustainable diet, including an increase in local and seasonal produce, reduction in food waste, and an increase in plant-based food intake [36]. This reflects on the contemporary nature of the research and the recent introduction of sustainable diets in the field of public health nutrition. The remaining studies focused on one or two sustainable diet outcomes only, such as an increase in fruit and vegetable intake (*n* = 18, 82%), reduction in discretionary food consumption (e.g., EDNP) (*n* = 2, 9%), and a decrease in red meat intake (*n* = 2, 9%). Because of this variation, the effectiveness of interventions on promoting sustainable diets comprehensively to young adults may be limited.

Most interventions aimed to increase fruit and vegetable intake of which most interventions were effective. However, out of all plant-based foods, the sole focus was on fruits and vegetables and not much evidence was found on other plant-based foods, such as whole grains and legumes. A previous systematic literature review which synthesised evidence on behavioural interventions aiming to promote more plant-based and less animal-based diets showed similar findings with respect to promoting fruits and vegetables as plant-based foods only [38]. The review suggested that future interventions may need to promote other plant-based foods, such as whole grains and legumes that are highly nutritious, and not only focus on fruits and vegetables [38]. In addition, this is especially important to consider because plant-based meat substitutes are becoming more popular and more integrated in people’s diets by the day [38].

The positive effects of sustainable diet outcomes were measured mainly on the short-term or only post-intervention, while studies showing long-term effectiveness (12 months or longer) were scarce (5 out of 22 studies) [21,23,24,25,30]. Only two studies have shown the intervention to be effective over the long-term (15- and 12-month follow-ups, respectively) in increasing fruit and vegetable intake [24,30]. The intervention by Green et al. [24] consisted of a 10-lesson online curriculum over a 10-week period targeting nondiet principles. Whereas in Nitzke et al. [30], the intervention included mailed educational materials and educational phone calls regarding fruit and vegetable intake over six months. Both studies employed the Transtheoretical Model and were classified as strong quality, although they differed greatly in intervention design and length. Thus, researchers may consider integrating longer follow-up measurement times in future research to provide better evidence regarding long-term maintenance of behaviour change. This finding is consistent with previously conducted systematic literature reviews, which highlighted the need to investigate the long-term effects of interventions showing effectiveness in improving eating behaviours [14,39]. These reviews also looked at effectiveness of dietary interventions for promoting healthy eating among university students and adults [14,39].

It was encouraging that a majority of studies (17 out of 22) employed behaviour change theories into intervention design and development. Theory-based interventions conduct the study development and evaluation process based on a framework and provide a better understanding of factors that lead to behaviour change [40]. Of the studies based on theory, 83% resulted in positive outcome changes. One of the most common theories was the Transtheoretical Model, which has been recognized as highly effective in changing dietary behaviours [41]. Other behaviour change theories, such as Theory of Planned Behaviour, Social Cognitive Theory, and Self-Affirmation Theory, were also linked with improved dietary outcomes. Therefore, it is still unknown if the choice of behaviour change theory(s) leads to greater intervention effects. A similar finding from a recent systematic review showed more research is needed to identify which behaviour change theory(s) contribute to greater intervention effectiveness [10].

A limited number of studies performed program delivery evaluation (8 out of 22), in terms of participants’ feedback, overall enjoyment, satisfaction, and engagement with the web-based interventions. Studies which evaluated such outcomes have been shown to be mainly positive, which can be an indicator of the usefulness of online platforms in promoting sustainable and healthy diets among young adults. These findings align with previous research indicating higher effectiveness in web- or media-based interventions compared to face-to-face delivery [14]. Interestingly, no studies were found that used social media platforms (Twitter, Facebook, Instagram, etc.) for program delivery, which is unusual given that young adults are frequent users [41]. One study used mobile phone texting to deliver behaviour-directed motivational messages aiming to increase fruit and vegetable intake [12]. During the program evaluation, participants voted for text messaging followed by Facebook as the preferred platforms for receiving nutrition-related content [12]. This suggests that future nutrition interventions for young people could consider social media platforms as a cost-effective, wide-reaching, and user-appropriate delivery mode compared to face-to-face interventions [15,41,42].

### Strengths and Limitations

There are some strengths and limitations to this review, which should be acknowledged. Whilst sustainable and healthy diet is an emerging concept in the field of public-health nutrition, the most important strength about this review is that it is the first to explore sustainable dietary outcomes in nutrition interventions. Another strength is that this review included a high proportion of RCTs (77% of studies), and RCTs are expected to maximize reliability of study effects and reduce risk of bias.

This review also had some limitations. For instance, this review was limited to articles published in the English language, and there may have been other relevant articles which were not included. It is important to note that most studies included a majority of female participants, which may have resulted in gender biases in findings. In addition, almost all study participants were university or college students recruited through their educational institutions. Only two studies recruited participants from noncollege or public settings, which might affect the representation of the target population. Future research targeting young adults might need to develop more varied and inclusive recruitment channels that would also enhance generalizability of the results to the broader young adult population. For instance, Kerr et al. [26] used the Federal Electoral Roll for recruitment, a compulsory enrollment system for Australians over 18 years old [26]. Intervention delivery lengths varied widely among studies. Some interventions were delivered over 30–45 min [23,32], some others over one to seven weeks [12,19,20,21,27,29,35,36], or two to six months [11,13,22,24,25,26,28,30,33,34,37]. Because of this large variation in interventions, delivery length, and follow up, it was challenging to provide conclusive evidence regarding the duration and intensity of effective interventions in promoting sustainable dietary behaviours.

## 5. Conclusions

Overall, this systematic literature review showed that there is potential for web-based nutrition interventions to promote sustainable and healthy diets among young adults. However, they were limited studies, which targeted more than one component of sustainable diets with the majority of studies focusing on increasing fruit and vegetable consumption. Sustainable and healthy diets are a multidimensional concept; therefore, there is a need for the development and evaluation of more high-quality and comprehensive web-based interventions aiming to promote sustainable diets. Only a few studies included long-term/follow-up measures, which highlights the need to investigate the effectiveness of interventions over time. With previous research indicating the potential impact of social media use in improving dietary behaviours in young adults, researchers could consider different platforms, such as Facebook and Instagram, for future web-based interventions.

## Figures and Tables

**Table 1:** Inclusion and exclusion criteria for systematic review of web-based interventions to promote sustainable and healthy diets in young adults.

	Inclusion	Exclusion
Population	Young healthy adults (18–25) except who are listed in the exclusion criteria	Young adults diagnosed with a noncommunicable disease or any other medical condition(s), which requires following a specific diet, or are outside the age range specified
Intervention	Web-based interventions promoting sustainable diets in at least one of its different aspects (e.g., increasing consumption of plant-based foods, decreasing food waste, reducing red meat consumption and discretionary foods)	Non-web-based/non-online-based interventions Web-based weight loss interventions
Comparator	No- or minimal-intervention controls and preintervention baseline	-
Outcome	Knowledge and food preparation skills about sustainable and healthy diet Intentions and attitudes in consuming sustainable and healthy diet Adherence to sustainable and healthy diet (reduced red meat and processed food intake, increase in fruit and vegetable intake, reduced food waste) Program delivery outcomes: engagement, retention, acceptability, compliance	General diet quality score only Studies measuring delivery of the program only
Study design	Randomized controlled trials (RCTs) Nonrandomised controlled trials Studies with a pre- and post-design	All other study designs that are not listed in the inclusion criteria

Strong—1; Moderate—2; Weak—3; ** Overall: Strong—1 (no WEAK rating), Moderate—2 (one WEAK rating), Weak—3 (two or more WEAK ratings).

**Figure A1:**
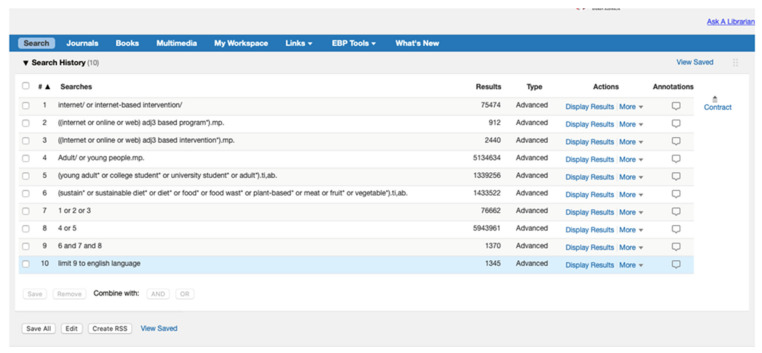
Medline search strategy.

## Data Availability

Not applicable.

## Supplementary Materials

The following supporting information can be downloaded at: https://www.mdpi.com/article/10.3390/ijerph19031691/s1, Table S1: Characteristics of included individual level studies (*n* = 22); Table S2: Outcomes and main findings of included individual level studies (*n* = 22).

## Appendix A

Search strategy

Keywords and linking words were used as follows:

1. Internet/ or Internet-based intervention/
2. ((Internet or online or web) adj3 based program*).mp.
3. ((Internet or online or web) adj3 based intervention*).mp.
4. Adult/ or young people.mp.
5. (young adult* or college student* or university student* or adult*).ti,ab.
6. (sustain* or sustainable diet* or diet* or food* or food wast* or plant-based* or meat or fruit* or vegetable*).ti,ab.
7. 1 or 2 or 3
8. 4 or 5
9. 6 and 7 and 8
10. Limit 9 to English language

The following is an example of the Medline search:

The same search strategy was repeated on PsychINFO, Embase, and Scopus.

## Appendix B

**Table A1:** Quality assessment of included studies.

Author(s) (Year), Country	Selection Bias	Design	Confounders	Blinding	Data Collection Methods	Withdrawals and Drop Outs	Overall
**Brown et al., 2011, USA** [34]	Moderate	Weak	Weak	Weak	Moderate	Moderate	Weak
**Cameron et al., 2015, UK** [13]	Moderate	Strong *	Strong *	Strong *	Strong *	Weak	Moderate
**Carfora et al., 2017a, Italy** [19]	Moderate	Strong *	Weak	Moderate	Moderate	Moderate	Moderate
**Carfora et al., 2017b, Italy** [20]	Moderate	Strong *	Weak	Moderate	Moderate	Strong *	Moderate
**Duan et al., 2017, Hong Kong** [22]	Moderate	Strong *	Weak	Strong *	Strong *	Weak	Weak
**Epton et al., 2014** [11]	Weak	Strong *	Weak	Strong *	Weak	Moderate	Weak
**Fielden et al., 2016** [35]	Weak	Moderate	Strong *	Moderate	Moderate	Strong *	Moderate
**Franko et al., 2008, USA** [23]	Moderate	Strong *	Strong *	Strong *	Strong *	Strong *	Strong **
**Greene et al., 2012, USA** [24]	Moderate	Strong *	Strong *	Strong *	Strong *	Moderate	Strong **
**Kattelmann et al., 2014, USA** [25]	Weak	Strong*	Strong *	Strong *	Moderate	Weak	Weak
**Kypri et al., 2005, New Zealand** [27]	Moderate	Strong *	Weak	Strong *	Moderate	Strong *	Moderate
**Park et al., 2008, USA** [32]	Moderate	Strong *	Strong *	Moderate	Strong *	Strong *	Strong **
**Whatnall et al., 2019, Australia** [33]	Moderate	Strong *	Moderate	Strong *	Moderate	Moderate	Strong **
**Nitzke et al., 2006, USA** [30]	Strong	Strong *	Strong *	Strong *	Strong *	Moderate	Strong **
**Clifford et al., 2009, USA** [21]	Moderate	Strong *	Strong *	Strong *	Weak	Weak	Weak
**Brown et al., 2014, USA** [12]	Moderate	Moderate	Strong *	Strong *	Weak	Strong *	Moderate
**Richards et al., 2006, USA** [37]	Moderate	Strong *	Strong *	Moderate	Weak	Moderate	Moderate
**Kerr et al., 2016, Australia** [26]	Moderate	Strong *	Strong *	Strong	Weak	Strong *	Moderate
**Lachausse, R. G. (2012), USA** [28]	Moderate	Strong *	Weak	Weak	Weak	Strong *	Weak
**Meng, J. et al. (2017), USA** [29]	Weak	Strong *	Strong *	Strong *	Weak	Moderate	Weak
**Monroe, J. T. et al. (2015), USA** [36]	Strong *	Moderate	Weak	Moderate	Strong *	Strong *	Moderate
**O’Brien, L. M., Palfai, T. P. (2016), USA** [31]	Moderate	Strong *	Strong *	Strong *	Strong *	Strong *	Strong **

* Strong—1; Moderate—2; Weak—3; ** Overall: Strong—1 (no WEAK rating), Moderate—2 (one WEAK rating), Weak—3 (two or more WEAK ratings).
