# Supplementary material for: Investigating Web-Based Nutrition Education Interventions for Promoting Sustainable and Healthy Diets in Young Adults: A Systematic Literature Review
Source: Int J Environ Res Public Health. 2022 Feb 1;19(3):1691. doi: 10.3390/ijerph19031691 (PMC8835600; doi:10.3390/ijerph19031691)
Supplement: Supplementary file 1 [file ijerph-19-01691-s001.zip › ijerph-1537161-supplementary.pdf]

Table S1. Characteristics of included individual level studies (*n* = 22).

Author(s) (year), country	Sample characteristics (Participant status, Mean age (SD), Gender)	Sample size	Intervention description	Delivery mode	Intervention length	Behaviour change theory/ Framework	Sustainable diet outcomes measures
**Randomized controlled trials (RCTs)**							
**Cameron et al., 2015 United Kingdom**	Undergraduate students Mean age: ≈ 18-19 Female: 55.34%	2621	IG: "*U@Uni: LifeGuide*" intervention including self-affirmation manipulation, health messages and implementation intention tasks to promote four health behaviours: F&V intake, smoking status, PA, and alcohol consumption. CG: measurement-only group.	Website & mobile application	6 months	TPB & SAT	• F&V serves • Self-efficacy of F&V • Program engagement
**Carfora et al., 2017a Italy**	Undergraduate students Mean age: 19.37 (±1.55) Female: 56%	112	IG: daily SMS on *Whats App* focusing on anticipated regret and the urge to self-monitor PMC based on encouragement of written self-monitoring of behaviour. CG: no intervention.	Text-messaging	1 week	TPB	• PMC (portions/week) • Intensions, affective and instrumental attitudes, and anticipated regret on PMC
**Carfora et al., 2017b Italy**	Undergraduate students Mean age ≈ 19.29 Female: 71.5%	228	IG: daily SMS which focused on anticipated regret and the urge to self-monitor RMC. CG: no intervention.	Text-messaging	1 week	TPB	• RMC (portions/week) • intentions, affective and instrumental attitudes, subjective norms on RMC
**Clifford et al., 2009 United States**	University students Mean age: NR Female: 63%	101	IG: the "*Good Grubbin'*" intervention consisting of four 15-minute episodes theory-based cooking show aiming to increase F&V intake. CG: no intervention.	TV cooking show	1 month	SCT	• F&V intake in cups • Knowledge regarding F&V • Attitudes and behaviour (motivation, barriers, self-efficacy, and frequency) regarding cooking and consuming F&V. • Program feedback
**Duan et al., 2017 Hongkong China**	University students Mean age: 19.3 (± 1.07) Female: 53.75%	493	IG: website targeting social-cognitive indicators for health behaviour change (e.g., risk perception, goal setting, action plans) to improve PA and F&V intake CG: no intervention.	Website	8 weeks	HAPA	• F&V intake (serves) • Self-efficacy in F&V intake

**Epton et al., 2014 United Kingdom**	First year university students<br>Mean age: 18.9<br>Female: 58%	1445	IG: *"U@Uni"* study targeting four health behaviours (F&V intake, smoking, alcohol intake and PA). Tasks for IG included self-affirmation manipulation (personal values) and access to all website educational materials (text, videos, etc.) regarding their health behaviour of interest. Educational materials included information and instructions regarding the 4 health behaviours and implementation intentions towards behaviour change. CG: no intervention.	Website & mobile application	6 months	SAT & TPB	• F&V intake (portions per day)<br>• Social cognitive variables (self-efficacy, intention, and attitude)<br>• Overall engagement
**Franko et al., 2008 United States**	Undergraduate students<br>Mean age: 20.1 (± 1.7)<br>Female: 56.3%	476	IG1: "*MSB-N"* web-based program comprised of three information links; rate myself assessment; four main topic pages, which contained text-based and audio information, interactive activities, and goal setting areas; and resources.<br>IG2: MSB-N + booster session<br>CG: anatomy education website	Website	IG1: 2 45min web-session for 2weeks<br><br>IG2: 3 45min web-session for 5 weeks	TTM	• F&V intake (serves)<br>• Self-efficacy for F&V intake<br>• Readiness to increase F&V intake
**Greene et al., 2012 United States**	College students<br>Mean age: 19.1 (± 1.1)<br>Female: 62%	1689	IG: Project "*Webhealth"* consisting of 10 lessons targeting healthful eating and PA through mini quizzes, graphs, charts, cartoons, and research findings.<br>CG: no intervention	Website	10 weeks	TTM & SCT	• F&V intake (cups)
**Kattelmann et al., 2014 United States**	College students<br>Mean age: 19.3 (± 1.1)<br>Female: 70.4%	1639	IG: *"Project YEAH"* comprised of 21 mini-educational lessons and e-mail messages to improve F&V intake, self-instruction, and self-regulation of healthful mealtime behaviour.<br>CG: no intervention.	E-mails	10 weeks	TTM	• F&V intake (cups)<br>• Program acceptance
**Kerr et al., 2016 Australia**	Young adults<br>Mean age ≈ 24.2-25<br>Female: 64.3%	247	IG(A): "*mFR app"* dietary feedback regarding F&V intake, EDNP, and sugar sweetened beverages through food pictures and text messaging<br>IG(B): dietary feedback only.<br>CG: no intervention.	Text messaging & mobile application	6 months	SDT & MI	• F&V (serves)<br>• Energy-Dense Nutrient-Poor (EDNP)
**Kypri & McAnally, 2005 New Zealand**	University students<br>Mean age: 20.2(± 1.5)<br>Female= 49%	148	IG(A): assessment + feedback through a web-based primary care intervention for multiple health risk behaviours including F&V intake, alcohol consumption, PA and smoking.<br>IG(B)= assessment only.	Website	6 weeks	NA	• F&V intake (serves) based on dietary recommendations (% of participants complying)

			CG: no intervention.				
**Lachausse 2012 USA**	College students<br>Mean age: 24.85<br>Female: 75%	312	IG1(online course): "*My Student Body (MSB)*"-Nutrition program including 4 learning modules (Nutrition 101, eating on the run, weighing in and fitness).<br>IG2 (on-campus course) addressing weight management, eating behaviours and overall wellness.<br>CG: conditions were not specified	Website	12 weeks	NA	• F&V intake (portions per day)<br>• Self-efficacy in F&V intake
**Meng et al., 2017 USA**	Undergraduate students<br>Mean age: 19.99 (± 1.7)<br>Female: 67%	73	IG: online groups consisting of 1 participant and "confederates". Program included 3 modules: a group goal setting, group self-tracking for F&V intake and bar graph illustrating weekly summaries.<br>CG: individual self-tracking for F&V intake	Website	4 weeks	NA	• F&V intake (serves)
**Nitzke et al., 2007 United States**	Economically disadvantaged young adults (noncollege venues)<br>Age range: 18-24<br>Female: 61.2%	2042	IG: multi-modal TTM-based intervention including mailed educational materials and phone calls to improve F&V intake<br>CG: mailed pamphlet	Emails & 2 educational calls	6 months	TTM	• F&V intake (serves)<br>• Perceived daily intake: self-efficacy, stage of change, decisional balance, and processes of change
**O'Brien & Palfai, 2016 USA**	Psychology class students<br>Mean age: 19.2 (± 1.2)<br>Female: 51%	154	Two brief intervention approaches to help students attain recommended standards for F&V intake;<br>IG1-web-based intervention with added text messaging regarding health and fitness to support behaviour change (WB+M).<br>IG2-web-based intervention with integrated personalized feedback, motivation, and self-regulation strategies (WBO).<br>CG (AO): assessment of eating behaviours only	Website & text messaging	30 days	GST	• F&V intake (serves)
**Park et al., 2008 USA**	Young adults<br>Mean age: 20.4 (± 1.6)<br>Female: 78%	111	IG: "*F&V Express Bites*" module including submodules consisting of stage-appropriate quizzes, tips and techniques for success, video vignettes and nutrition messages.<br>CG: no intervention.	Website	20-30 minutes	TTM	• Self-efficacy and decisional balance for F&V intake<br>• Program applicability and usefulness

**Whatnall et al., 2019 Australia**	University students Mean age: 22.4 Female: 72.6%	124	IG: *"EATS"* brief web-based nutrition intervention for eating advice aiming to improve four eating behaviours (F&V intake, discretionary foods, breakfast). CG: existing web-based alcohol intervention.	Website	3 months	SCT & TPB	• F&V intake (serves) • Discretionary food intake • Self-efficacy for F&V intake Program acceptability
			**Pre- post-design studies**				
**Brown et al., 2014 United States**	University students Age: 22 (57%) Female: NR	116	IG: *"Mobile MyPlate"* text messages containing behaviour-directed motivational dietary guidelines messages CG: received a brochure at baseline with the MyPlate icon along with the same dietary guidelines messages.	Mobile phone texting	7 weeks	NA	• F&V intake • Program evaluation
**Brown, et al., 2011 United States**	University students Mean age: 20 Female: 58%	281	IG: *"The Viva Vegetables!"* program combining online video instruction on selecting, storing, and preparing target vegetables to increase self-efficacy for vegetable preparation through an online, skill-based intervention.	Online videos & in-class tasting experience	4 months	TTM	• Vegetable intake (serves) • Self-efficacy for vegetable preparation
**Fielden et al., 2016 United Kingdom**	Undergraduate students Mean age: 21.6 (± 3.2) Female: 100%	59	IG: health messages concerning F&V intake based on self-affirmation (SA) manipulation in two high risk of low intake groups: young adults and low SES mothers. CG: Non-Affirmed (NA).	Website	7 days	SAT	• F&V intake (portions)
**Monroe et al., 2015 United States**	University students Mean age: 18.88 Female: 77.8%	608	IG: "*The Green Eating (GE)*" project promoting environmentally conscious eating behaviours included 4 modules (Introduction to GE, EAT local, reduce waste, environmentally friendly protein). CG: unrelated online survey online	Website	5 weeks	TTM	• Green eating (GE) stage of change, knowledge and GE behaviours • Self-efficacy decisional balance and in regard to GE. • Program evaluation
**Richards et al., 2006 USA**	University students Mean age: 20.4 (± 1.5) Female: 75.2%	314	IG: Stage-based newsletters, stage-based motivational interviewing, computer-based follow-ups, and a nutrition website to motivate young adults to consume more F&V over. CG: no intervention.	Website & Emails	4 months	TTM	• F&V intake (serves). • Self-efficacy in F&V intake

CG- control group; FFQ- Food Frequency Questionnaire; F&V – Fruits and vegetables; GST- Goals Systems Theory; HAPA- Health Action Process Approach; IG-intervention group; MI- Motivational Interviewing; NR- not reported; PA – Physical activity; PMC – Processed Meat Consumption; SAT- Self-Affirmation Theory; SCT- Social Cognitive Theory; SDT- Self-Determination Theory; TPB- Theory of Planned Behaviour; TTM- TransTheoretical Model; RMC – Red Meat Consumption; ±- standard deviation.

Table S2. Outcomes and main findings of included individual level studies (n = 22).

Author(s) (year), country	Outcomes	Tools	Timing of Measurement	Main findings	Overall quality assessment score
**Randomized Controlled trials (RCTs)**					
**Cameron et al., 2015 United Kingdom**	F&V intake (serves)	Two-item dietary questionnaire	Baseline, 1-month and post-intervention	F&V intake: significantly different at both timepoints for IG compared with CG; 1-month (IG mean= 3.84±1.95; CG mean= 3.63±1.84, p=0.041) and at 6-month (IG mean= 4.11±1.84; CG mean= 3.89±1.97, p=0.024).	Moderate
	Self-efficacy of F&V	Single-item measure		Self-efficacy FV: Participants in IG had lower self-efficacy for F&V intake than CG at 6-month follow-up.	
	Program engagement	% Participants completing the program's tasks		Engagement: 85% completed the self-affirmation task, 47% viewed health messages and only 29% formed implementation intentions for all four behaviours.	
**Carfora et al., 2017a Italy**	Processed Meat Consumption (PMC) (portion/week)	Online food diary	Baseline and post-intervention (T2)	The intervention was effective in increasing intentions to reduce and reducing intake of PMC. PMC: IG ate significantly fewer servings (mean= 1.74) than the CG (mean= 3.29, p< 0.001).	Moderate
	Intensions, affective and instrumental attitudes, and anticipated regret on PMC	Questionnaire		At post- intervention, the IG reported higher intentions to eat ≤1 serving of processed meat in the upcoming week (mean = 4.47) compared to the CG (mean = 3.60, p < 0.008).	
**Carfora et al., 2017b Italy**	Red Meat Consumption (RMC) (portion/week)	Online food diary	Baseline and post-intervention (T2)	The intervention was effective in increasing intentions and reducing RMC. During the intervention the IG ate significantly fewer servings of red meat (mean = 1.62) than the CG (mean =3.03, p<0.001).	Moderate
	Intensions, affective and instrumental attitudes, subjective norms on RMC	Questionnaire		At post- intervention, the IG reported higher intentions to eat < 2 servings of RMC over the upcoming week (mean = 4.80) compared to the CG (mean = 4.07, p < 0.01).	
**Clifford et al., 2009 United States**	F&V intake (cups)	FFQ	Baseline, post-intervention and 4-month follow-up	F&V intake: no significant differences between the IG and CG.	Weak
	Knowledge regarding F&V intake	Questions based on dietary guidelines		F&V knowledge: significant improvements in in the IG compared to the CG postintervention and at follow-up (p<0.0001).	
	Attitudes and behaviour (motivation, barriers, self-	5-point likert scale		Attitudes and behaviour: IG experienced a significant improvement in cooking motivators, barriers (p <0.05), but not in self-efficacy	

	efficacy, and frequency) regarding cooking and consuming F&V			(p<0.1) from pre to post compared to the CG. This improvement was not maintained at follow-up.<br>There were no significant changes in, motivators, barriers, and self-efficacy in the IG compared to CG.	
	Program feedback (overall enjoyment and interest)	Survey		Program feedback: 80% of viewers enjoyed the program.	
**Duan et al., 2017 Hongkong China**	F&V intake (serves)	4-item short questionnaire	Baseline, post intervention, and at 1-month follow-up	F&V intake: the average amount in IG were all greater than the recommended amounts (5 serves per day) at baseline (6.3 portions) and at post-intervention (5.8 portions).	Weak
	Self-efficacy in F&V intake	5-point scale questions		Self-efficacy in F&V intake: significant stage progression to the action group from baseline to post-intervention (p=0.03).	
**Epton et al., 2014 United Kingdom**	F&V intake (portions per day)	Health Survey for England (HSE)	Baseline, 1 month follow-up and post-intervention (6 months)	F&V intake: no significant differences between IG and CG. Mean portions at 1-month follow-up: CG mean= 5.47, IG mean= 6.02 p=0.053, and mean portions at 6-month follow-up: CG mean= 5.72, IG mean= 5.61 p=0.708.	Weak
	Social cognitive variables (self-efficacy, intention, and attitude)	1 or 2 item(s) questions		Social cognitive variables: self-efficacy, intention, and attitude towards F&V intake, no significant changes were reported at 1-month and 6-month follow-up.	
	Overall engagement	% Participants completed the program's tasks behaviour		Overall engagement: 52% of participants completed the self-affirmation task, 35% accessed the health messages, only 1% made an implementation plan and only 2% downloaded the app. The overall engagement with the intervention was low.	
**Franko et al., 2008 United States**	F&V intake (serves)	FFQ (16 items) and a single-item measure	Baseline, post-test, 3-month, and 6-month follow-up.	F&V intake: significant increase in IG1 and IG2 by 0.32 and 0.24 servings, respectively, relative to the CG at post-test (p < 0.01) but not at 3 or 6-month follow-up.	Strong
	Self-efficacy for F&V intake	3-item questionnaire		Self-efficacy for F&V intake: IG1 and IG2 were more likely to increase their self-efficacy (p< 0.05) compared to CG.	
	Readiness to increase F&V intake	14-item questionnaire		Readiness to increase F&V intake: IG1 and IG2 were more likely to improve readiness to increase F&V intake than CG at post-test (p< 0.0001). Only IG1 was more likely than CG to advance in readiness to increase F&V intake at 6-month follow-up (p< 0.05).	
**Greene et al., 2012 United States**	F&V intake (cups)	Two-item screener and the National	Baseline, 3-month post-intervention and 15-month follow-up	F&V intake: IG significant increase by 1 cup from baseline to postintervention [(Two-item screener: baseline= 2.6±0.10; post-	Strong

		Cancer Institute (NCI) F&V screener		intervention=3.7±0.10); NCI: baseline = 3.3±0.12; post-intervention= 4.1±0.16)]. CG decreased their intake by 0.1 to 0.3 cups per day [(Two-Item F&V: baseline = 2.6± 0.09; post-intervention= 2.5± 0.10); (NCI F&V: baseline =3.1± 0.12; post-intervention= 2.8± 0.15)]. Over 15 months, IG had significantly higher F&V intake (0.5 cups/day).	
**Kattelmann et al., 2014 United States**	F&V intake (cups)	National Cancer Institute F&V Screener (short form)	Baseline, postintervention (10 weeks) and 15-month follow-up	F&V intake (cups): IG reported small but statistically significant increases in cups of total from baseline 2.6±2.1 cups to postintervention 2.8±2.1 cups, whereas the CG decreased cups of F&V, resulting in a significant group difference with a small effect size (Cohen d = 0.05) between IG and CG at postintervention. F&V intake was not maintained at follow-up.	Weak
	Program acceptance	Online survey		Program acceptance: 57% of participants completed the process evaluation survey, of these 87% rated the program as good or excellent.	
**Kerr et al., 2016 Australia**	Fruit, vegetable, and Energy-Dense Nutrient-Poor (EDNP) food intake (serves)	4-day food record (food pictures)	Baseline and post-intervention	Vegetable intake: IG(B) and CG increased significantly (p=0.002) post-intervention, but not in IG(A)	Moderate
				Fruit intake: IG(A) significant increase (p=0.03).	
				EDNP serves significant decrease in both IG(A) and IG(B) (-0.8±0.2; p <0.001) post-intervention.	
**Kypri & McAnally, 2005 New Zealand**	F&V intake (serves) based on dietary recommendations (% of participants complying)	Two questions from New Zealand National Survey questionnaire	Baseline and post-intervention.	IG(A) had significantly greater compliance with recommendations than did CG (IG(A)= 33% vs IG(B)=26% vs CG=13%). Differences between IG(A) and IG(B), and IG(B) vs. CG were non-significant post-intervention.	Moderate
**Lachausse 2012 USA**	F&V intake (portions per day)	FFQ based on the Center for Disease Control and Prevention's Youth Risk Behaviour Survey	Baseline and post-intervention	Fruit intake: frequency of consumption significantly increased from baseline (mean = 2.67±1.25) to post-intervention (mean = 3.37±1.60) for IG1. For vegetable intake, the frequency significantly increased from baseline (mean = 2.44±1.22) to post-intervention (mean= 2.80±1.35) for the online group (IG1) but not in the on-campus (IG2) or CG	Weak
	Self-efficacy in F&V intake	F&V Self-Efficacy Scale		F&V self-efficacy: significant increase from baseline to post-intervention in IG1 (p= 0.001), but not in the on-campus (IG2) or CG.	

**Meng et al., 2017 USA**	F&V intake (serves)	FFQ	Baseline and post-intervention	F&V intake: significant increase in IG (group-based self-tracking) (mean=3.37±2.01) compared to CG (individual-based self-tracking) (mean= 1.37±1.44) (p=0.01).	Weak
**Nitzke et al., 2007 United States**	F&V intake (serves)	5 A Day Screener (seven open-ended questions)	Baseline, 4 months, and 12-month follow-up	F&V serves/day: significant increase in IG was observed compared to CG; 4.35± 3.15 and 4.02±3.18 respectively (p<0.05) at 12 months follow-up.	Strong
	Perceived daily intake based on self-efficacy, stage of change, decisional balance, and processes of change	Short likert-scale questions		Perceived daily intake: IG had significantly higher perceived intake of F&V than CG; with daily intake of 4.96 (± 2.59) and 4.75 (± 2.68) respectively (p<0.05)	
**O'Brien & Palfai, 2016 USA**	F&V intake (serves)	Modified dietary habits questionnaire consisting of three 7-day items based on the large-scale CDC Youth Risk Behaviour Surveillance system (YRBS)	Baseline and post-intervention	Significant benefits of the WB+M intervention on attainment of vegetable intake guidelines compared to controls; (IG1= 37%; IG2=22%; CG=14% [aOR] = 2.93, 95% CI: 1.06, 8.12, p = 0.04). Non-significant benefits of the WB+M intervention on attainment of fruits intake guidelines compared to controls were reported; IG1 and CG (aOR = 1.54, 95% CI: 0.56, 4.26, p = 0.41) and IG1 and IG2 (aOR = 1.57, 95% CI: 0.55,4.26, p=0.37).	Strong
**Park et al., 2008 USA**	Self-efficacy and decisional balance for F&V intake	5 and 8 Likert-scaled questions	Baseline, post-intervention, and 30-60 days follow-up	Vegetable intake: significant progress to action/maintenance stages indicating self-assessed intake of ≥3 servings per day (p=0.039) in IG but not in CG (p=0.267). Fruit intake: participants were already in action/maintenance phase for fruit intake; no significant change detected (only marginal possible trend) (p=0.057) for IG but significant in CG (p=0.016).	Strong
	Program applicability and usefulness	26-item evaluation form		Mostly, participants feedback about the intervention was positive. Mean scores were most positive for understandability and organization, followed by design, recipes, quizzes, and video segments (4.33, 4.28, 4.11, 3.91, 3.19, and 2.77, respectively).	
**Whatnall et al., 2019 Australia**	F&V intake (grams/day)	Australian Eating Survey (AES) FFQ (120 items)	Baseline and post-intervention	F&V intake: no significant changes in both fruit (IG: p=0.999; CG: p=0.525) and vegetable intake (IG: p=0.332; CG: p=0.650) were reported.	Strong

	Discretionary food intake (serves)	Australian Eating Survey (AES) FFQ (120 items).		Discretionary food intake: Significant difference (−4.8%, 95%CI: −8.6, −1.1, p=0.012) was reported in favour of the IG.	
	Self-efficacy for F&V intake	Questions based on confidence in achieving behaviour change		Self-efficacy for F&V intake: significant increase in CG only (0.3 CI: 0.1, 0.6 p=0.002).	
	Program acceptability (usefulness, relevance, usability, and ability to motivate)	5-point likert scale questions		Program acceptability: overall positive with mean=4±0.7 for satisfaction and mean=4±0.8 for meeting expectations.	
**Pre- and post-design studies**					
**Brown et al., 2014 United States**	F&V intake was assessed using	FFQ	Baseline and post-intervention	Fruit intake: significant increase in the IG (p<0.05) Vegetable intake: a trend towards an increase.	Moderate
	Program evaluation (feedback and suggestions)	Post-intervention survey		Program evaluation: 98% of participants reported that the text messaging was a good idea to receive health information. Almost half (48%) of participants agree that text messages were the best way to communicate health information followed by Facebook (23%).	
**Brown, et al., 2011 United States**	Vegetable intake (serves)	Harvard Nurse's Study FFQ (123 food items)	Pre- and post-intervention	Vegetable intake: average total intake did not change from the baseline (1.63±1.24) to the post-intervention (1.63±1.3), p=0.980.	Weak
	Self-efficacy for vegetable preparation	5-point Likert scale		Self-efficacy: positively correlated with vegetable intake (R= 0.26; p =0.0001). Self-efficacy of vegetable preparation at the baseline was associated with increased total vegetable intake but not target vegetable intake (p=0.002 and p=0.065), respectively. The percent of participants who reported knowing how to prepare vegetables in many ways increased from 59.2% to 73.1% from the baseline to post-intervention (p =0.001).	
**Fielden et al., 2016 United Kingdom**	F&V intake (portions)	24-hour recall dietary	Baseline and post-intervention	F&V intake: IG reported consuming significantly more portions of F&V (SA: mean= 3.96±0.79, NA: mean = 2.81 ±1.3).	Moderate
**Monroe et al., 2015 United States**	Green eating (GE) stage of change, knowledge and GE behaviours	Survey	Baseline and post-intervention	GE behaviours: significant improvement in IG from baseline to post-intervention form (mean=2.33±0.80 to mean=2.60±0.81) compared to non-significant change in CG from (mean=2.45 ±0.81) to (mean=2.47 ±0.85). Knowledge in GE: IG significantly increased but not in CG. IG knowledge increased from baseline (mean=8.02 ±2.24) to post-intervention (mean=10.16 ±2.52) compared to CG; from (mean=7.82 ±2.22) to (mean=7.91 ±2.47)	Moderate

	Self-efficacy and decisional balance in regard to GE	5-point anchored Likert-scale questions		Self-efficacy and decisional balance in regard to GE: effective increase	
	Program evaluation	5-Likert scale questions		69.1% rated the program as motivational and 77.1% had a good or excellent final opinion	
**Richards et al., 2006 USA**	F&V intake (serves)	1-item FFQ and a 26-item FFQ	Baseline and post-intervention	F&V intake: IG increased by 1.0±0.1(p <0.001) and 1.0±0.3 (p=0.04) servings a day based on the 1-item and 26-item FFQ from baseline to postintervention respectively, compared with CG: 0.4±0.1 servings a day (p<0.001). Pre-action IG had a significantly greater increase in F&V intake for both 1-item and the 26-item FFQ than CG.	Moderate
	Self-efficacy in F&V intake	Five-item questionnaire		Self-efficacy scores for F&V were significantly greater in IG than CG at post-intervention. No significant difference in decisional balance for both IG and CG was observed at baseline and post-intervention.	

CG- control group; CI- Confidence Interval; FFQ- Food Frequency Questionnaire; F&V – Fruits and vegetables; GE – Green Eating; IG-intervention group; NA - Non-Affirmed; PA – Physical activity; PMC – Processed Meat Consumption; RMC – Red Meat Consumption; SA – Self-Affirmed; ±- standard deviation.
